# Ultrasound-guided percutaneous screw fixation of sternal metastasis

**DOI:** 10.1016/j.radcr.2025.02.056

**Published:** 2025-03-15

**Authors:** Thibaud Morcet-Delattre, Thibaut Affole

**Affiliations:** Department of Interventional Radiology, Centre de Lutte Contre le Cancer Eugène Marquis, Rennes, France

**Keywords:** Sternum, Bone screw, Ultrasound, Interventional radiology

## Abstract

Sternal metastasis often result in disabling pain, significant functional limitations, with potential consequences for the thoracic spine. This case report suggests a new approach combining ultrasound for initial guidance and fluoroscopy with cone-beam CT (CBCT) for screw fixation. The patient experienced immediate pain relief, a better mobility, and improved quality of life. This approach demonstrates a minimally invasive, radiation-sparing and time saving strategy for sternal screwing.

## Introduction

Sternal metastases, often originating from breast, renal, or lung cancer, frequently lead to painful fractures that significantly impair patient quality of life. These pathological fractures often require stabilization to alleviate pain and improve function [1].

While surgical resection remains the standard for isolated metastases, it is often invasive and associated with postoperative complications [[2], [3], [4], [5]]. Minimally invasive approaches, including percutaneous screw fixation, cementoplasty, and thermal ablation techniques such as radiofrequency ablation (RFA) and cryoablation, have gained traction due to their ability to provide pain relief, stabilize fractures, and improve patient function with reduced morbidity [[6], [7], [8], [9]]. Despite these advancements, there is limited data on the use of ultrasound guidance in percutaneous sternal fixation, which could provide an effective alternative by reducing radiation exposure and enhancing procedural accuracy

This case report describes a novel hybrid imaging approach that integrates ultrasound for initial trocar placement, followed by fluoroscopic and CBCT confirmation, in the percutaneous screw fixation of a sternal metastatic lesion. By presenting the technical feasibility, safety, and clinical outcomes, this study aims to highlight ultrasound guidance as a valuable addition to interventional radiology techniques for sternal metastases, offering a less invasive, radiation-sparing, and time-efficient alternative to conventional methods

## Case report

**Fig. 1:**
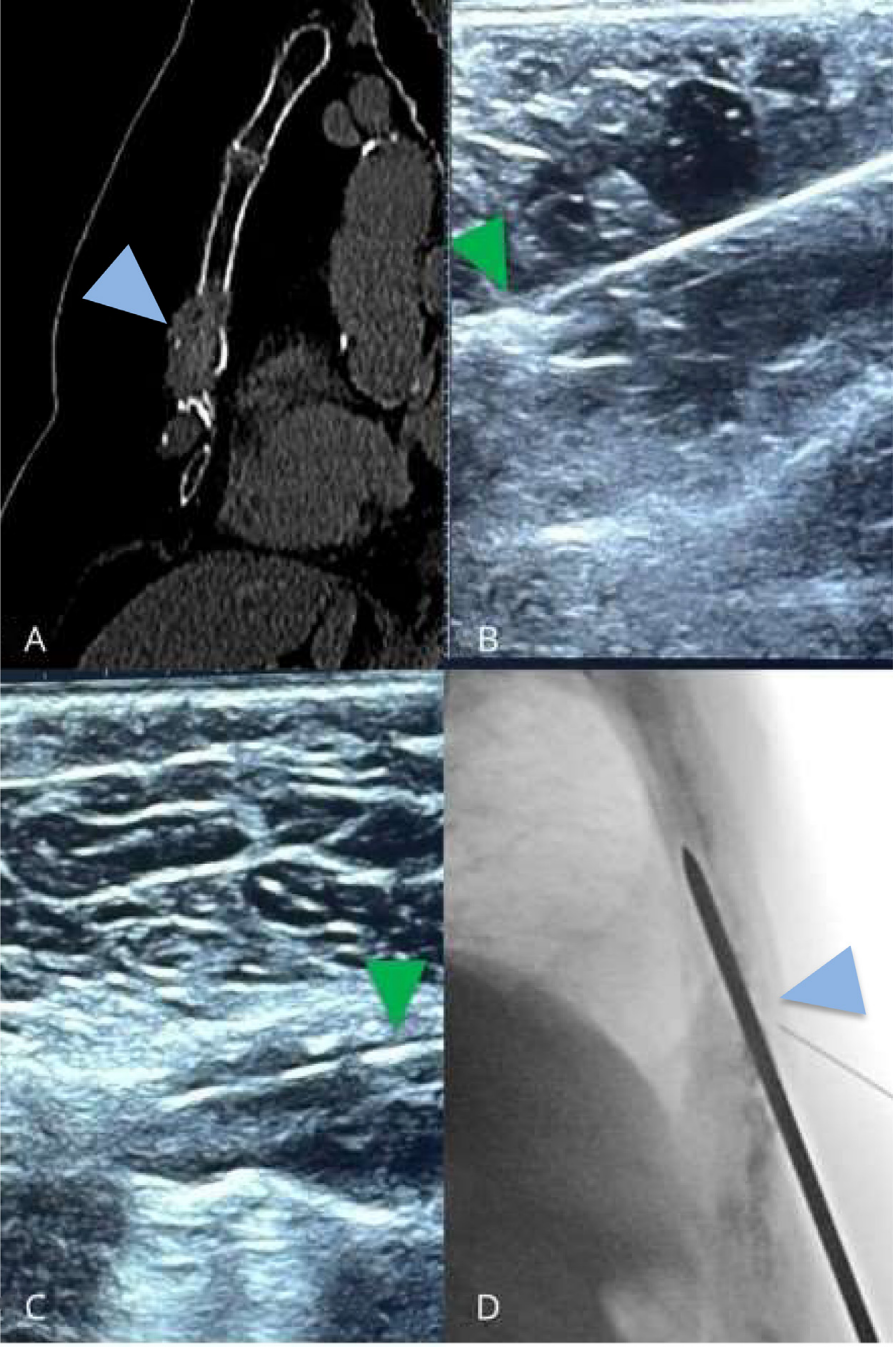
Diagnostic preoperative CT scan showing a large lytic lesion in the inferior half of the sternum with microfractures across the lesion (Blue Arrowhead). (A) 8G bone trocar visible under ultrasound guidance in contact with healthy bone (Green Arrowhead). (B) Visibility of the trocar into the lytic lesion under ultrasound (Green Arrowhead). (C) Lateral fluoroscopy confirming correct positioning of the trocar and 22G marker needle (Blue Arrowhead) (D).

The patient is a 71-year-old female with a history of metastatic renal cancer, diagnosed in 2015 with clear cell renal carcinoma and multiple metastases treated with surgery and immunotherapy. She has since experienced progressive metastatic disease involving the lungs, bones, and digestive complications. Despite multiple therapeutic adjustments (including nivolumab, cabozantinib, and axitinib) and stable pulmonary lesions, bone metastases, notably in the sternum, led to severe symptoms. The blood test revealed elevated monocytes (0.78 G/L; reference: 0.17-0.56) and creatinine clearance of 52 mL/min/1.73 m^2^, suggesting mild renal impairment and a potential inflammatory or immune-related process. All other parameters, including coagulation and thyroid function, were within normal ranges. The patient presented with severe chest pain localized to the lower sternum for 3 weeks despite analgesic treatment including opiod. The patient reported severe pain upon palpation of the sternum, which worsened with movement and breathing. Preoperative CT imaging revealed a lytic lesion in the lower half of the sternum, with healthy cortical remnant of the lower end of the sternal body (Fig. 1A). Following a multidisciplinary team discussion with spine surgeons, radiation oncologists, medical oncologists and interventional radiologists, it was decided to proceed with percutaneous screw fixation.

The procedure was performed under general anesthesia with the patient positioned in supine position. 2 g of Cefazolin were administrated intravenously.

**Fig. 2:**
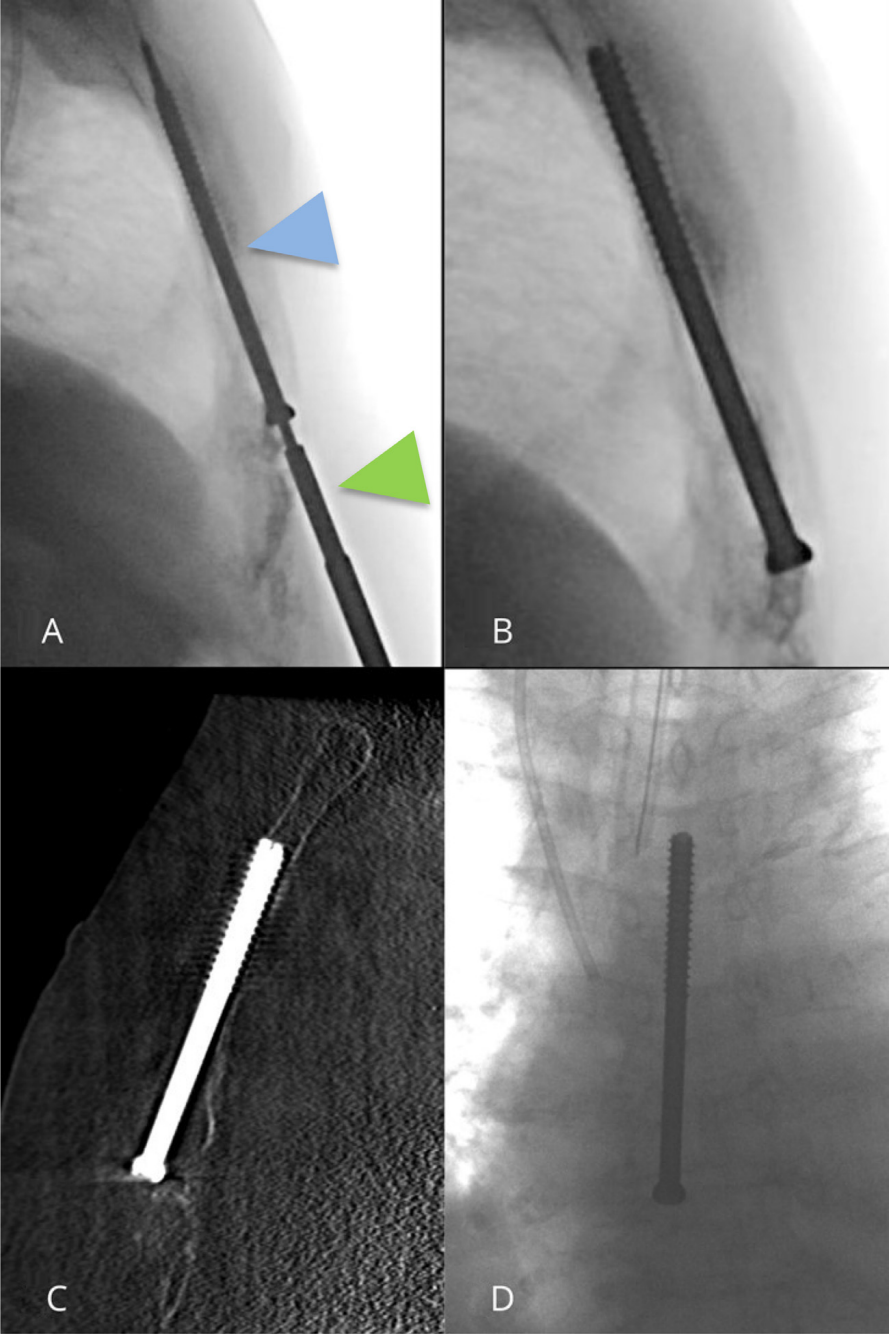
Lateral fluoroscopic control showing the partially threaded titanium Asnis III screw (Blue Arrowhead), on the Kirschner wire and the screwdriver (Green arrowhead) (A) Satisfactory screw position on fluoroscopy (B and D) Sagittal CBCT reconstruction showing the correct positioning of the screw (C).

The first step involved inserting of a 22-gauge spinal needle under ultrasound guidance (Acuson Freestyle, Siemens Healthineers, Princeton, NJ) up to bone contact with the remaining intact cortex. Confirmation of the needle position was achieved using cone-beam computed tomography (CBCT) (Artis Zee, Siemens Healthineers, Princeton, NJ). Once the bone entry point was confirmed, a 1 cm incision was made 7 cm below the target area to provide access to the inferior border of the sternum. This incision enabled the safe placement of an 8-gauge trocar bone (Stryker, Kalamazoo, MI), which was advanced under ultrasound guidance, through the tumor with good visibility until it reaches the remaining healthy bone medullary (Figs. 1B and C). After confirmation of the trocar's placement, the imaging modality was switched to lateral fluoroscopy and a 3.2 mm Kirschner wire (Fig. 1D) followed by a partially threaded titanium screw with a 6.5 mm diameter and 90 mm length (Asnis III, Stryker, Kalamazoo, MI) were inserted in the sternal body (Fig. 2). Iterative CBCT imaging was used throughout the procedure to monitor the screw's progression, ensuring precise placement within both the tumor and healthy bone while avoiding cortical breaches. The procedure was completed successfully without complications. Overall, the operating time was 40 minutes. The patient experienced immediate and total pain relief, with the Numeric Pain Rating Scale (NPRS) score decreasing from 7/10 preoperatively to 0/10 postoperatively. This was confirmed at the 1-month consultation with the interventional radiologist, with an overall improvement in the patient quality of life.

## Discussion

Sternal metastases are a complex clinical challenge, particularly when associated with pathological fractures that tend to deform under respiratory motion and heal poorly compared to other skeletal sites [1]. Furthermore, biomechanical studies suggest that the sternum plays a critical role in thoracic spine stability, with some authors referring to it as the “fourth column” of the axial skeleton [10].

Sternal resection remains a crucial option for isolated or oligometastatic disease, particularly in renal and thyroid cancers, where complete metastasectomy can improve survival [2,3]. Titanium mesh reconstruction provides durable thoracic support, reducing complications and enhancing recovery [4]. However, surgery carries risks, including postoperative infections and embolisms, as reported in cases of renal cell carcinoma metastasis [5]. Despite these challenges, multidisciplinary approaches integrating surgery, systemic therapy, and reconstruction techniques remain essential in optimizing patient outcomes [11]. Additionally, radiotherapy remains a cornerstone of metastatic sternal management, playing an important role in both pain control and tumor ablation [12].

Minimally invasive interventional radiology (IR) techniques, including embolization, percutaneous fixation, radiofrequency ablation (RFA), and cryoablation, have emerged as effective alternatives to surgery for pain control and structural stabilization in sternal metastases. Cementoplasty has long been used in nonvertebral metastatic bone lesions [[13], [14], [15]] and sternoplasty has been reported in multiple case studies, including one utilizing ultrasound and fluoroscopic guidance [14,[16], [17], [18], [19]].

Among these techniques, embolization has proven highly effective in reducing tumor vascularity and alleviating pain. In a study by Papalexis et al. [9], patients who underwent palliative arterial embolization experienced a 50% reduction in pain scores, along with a significant decrease in tumor size at 12-month follow-up.

Percutaneous fixation using internal cemented screws (FICS) has shown excellent pain control and structural stabilization, as seen in Deschamps et al. and Grange et al., where patients reported immediate postprocedural pain relief and improved quality of life [6,7]. RFA combined with cementoplasty, as explored by Masala et al. [8], offers a dual benefit of local tumor necrosis and mechanical reinforcement, achieving rapid symptomatic relief in a metastatic breast cancer case. Similarly, Hegg et al. [20] demonstrated that cryoablation provides durable pain control, with mean pain scores dropping from 7.0 to 1.8 and a local tumor control rate of 80%.

These findings highlight interventional radiology as a key modality for palliation and stabilization in patients with nonresectable sternal metastases, reducing dependency on surgery and systemic therapy while ensuring rapid functional improvement.

Ultrasound was utilized in this case to guide the placement of the spinal needle and the Stryker 8G trocar, significantly reducing radiation exposure during the initial phase of the procedure and shortening the overall intervention time compared to fluoroscopic-only methods. The real-time visualization provided by ultrasound allowed for accurate trocar placement in healthy bone, ensuring that the trajectory remained parallel to the sternal cortices while avoiding critical mediastinal structures.

The standard approach for metastatic sternal fractures typically involves internal cemented screw fixation (FICS). However, in this case, distal cementoplasty was not performed due to the presence of sufficient healthy bone for mechanical anchorage. Additionally, cement injection posed a high risk of leakage into the mediastinum, given the direct proximity of the tumor to adjacent structures.

While this approach presents several advantages, there are inherent limitations that must be acknowledged. The success of ultrasound-guided percutaneous fixation is highly dependent on operator expertise, making wider implementation challenging across institutions without trained specialists. Furthermore, advanced imaging techniques such as CBCT are not universally available, potentially limiting the reproducibility of this technique.

Future research should focus on comparing ultrasound-guided vs fluoroscopic/CBCT-only percutaneous fixation techniques in terms of radiation exposure, procedural efficiency, and technical accuracy, assessing long-term outcomes of percutaneous fixation vs surgical resection and exploring hybrid approaches (combining percutaneous fixation with tumor debulking techniques such as RFA or cryoablation, to achieve both structural and oncologic control).

These findings highlights the importance of a multidisciplinary, personalized approach in selecting the optimal treatment strategy for patients with sternal metastases, ensuring maximal pain relief, biomechanical stability, and improved quality of life

## Conclusion

This case illustrates the successful use of a combined imaging approach involving ultrasound, fluoroscopy, and iterative CBCT for the percutaneous screw fixation of a metastatic sternal fracture. Ultrasound was used to guide the initial entry and placement of the trocar, reducing radiation exposure while fluoroscopy and CBCT ensured precise screw placement. The patient experienced pain relief and improved mobility postprocedure, supporting the value of this technique. Further studies are needed to confirm the long-term outcomes of this treatment in a broader patient population.

## Ethics statement

Informed consent was obtained from the patient for the publication of this case report.

## Patient consent

I confirm that written, informed consent has been obtained from the patient for the publication of this case report, including all accompanying images and data. The patient has been fully informed about the nature of the report and has agreed to the use of de-identified information to ensure anonymity. No personal or identifying details have been included, and the patient understands that this case report will be publicly accessible.

The patient has provided written consent, which is securely stored at the Centre Eugène Marquis in Rennes, France, in accordance with institutional policies and ethical guidelines.
